# The Impact of Non-additive Effects on the Genetic Correlation Between Populations

**DOI:** 10.1534/g3.119.400663

**Published:** 2019-12-19

**Authors:** Pascal Duenk, Piter Bijma, Mario P. L. Calus, Yvonne C. J. Wientjes, Julius H. J. van der Werf

**Affiliations:** * Animal Breeding and Genomics, Wageningen University and Research, P.O. Box 338, 6700 AH Wageningen, the Netherlands and; † School of Environmental and Rural Science, University of New England, Armidale 2351 NSW, Australia

**Keywords:** dominance, epistasis, genomic prediction, average effects, population divergence

## Abstract

Average effects of alleles can show considerable differences between populations. The magnitude of these differences can be measured by the additive genetic correlation between populations ($r_{g}$). This $r_{g}$ can be lower than one due to the presence of non-additive genetic effects together with differences in allele frequencies between populations. However, the relationship between the nature of non-additive effects, differences in allele frequencies, and the value of $r_{g}$ remains unclear, and was therefore the focus of this study. We simulated genotype data of two populations that have diverged under drift only, or under drift and selection, and we simulated traits where the genetic model and magnitude of non-additive effects were varied. Results showed that larger differences in allele frequencies and larger non-additive effects resulted in lower values of $r_{g}$. In addition, we found that with epistasis, $r_{g}$ decreases with an increase of the number of interactions per locus. For both dominance and epistasis, we found that, when non-additive effects became extremely large, $r_{g}$ had a lower bound that was determined by the type of inter-allelic interaction, and the difference in allele frequencies between populations. Given that dominance variance is usually small, our results show that it is unlikely that true $r_{g}$ values lower than 0.80 are due to dominance effects alone. With realistic levels of epistasis, $r_{g}$ dropped as low as 0.45. These results may contribute to the understanding of differences in genetic expression of complex traits between populations, and may help in explaining the inefficiency of genomic trait prediction across populations.

Populations can differ considerably in the average effects of loci (*i.e.*, α, the difference between average effects of the two alleles, Falconer and Mackay (1996)). For a given genotype (*i.e.*, individual), differences in α between two populations lead to differences in the additive genetic values of that genotype, as expressed in both populations. The magnitude of these differences can be measured by the additive genetic correlation between populations ($r_{g}$), defined as the correlation between the additive genetic values of a genotype expressed in population 1 and population 2. In reality, a single genotype cannot belong to two populations at the same time. This means that a trait expressed in two populations can be seen as a pair of traits that cannot be measured on the same individual, analogous to *e.g.*, age at sexual maturity in males and females (Falconer and Mackay 1996). Although no phenotypic correlation exists between such pairs of traits, they can nevertheless be genetically correlated.

The $r_{g}$ can be lower than one due to genotype by environment interaction (GxE) (Falconer 1952), or due to non-additive genetic effects (GxG-interaction) together with differences in allele frequencies between populations (Fisher 1918). Knowledge of this correlation contributes to the understanding of the genetic architectures of polygenic traits (de Candia *et al.* 2013; Brown *et al.* 2016). Such understanding may lead to improved knowledge of genetics and can facilitate accurate prediction of traits, such as disease risk in humans and yield traits in crops (Forsberg *et al.* 2017). Furthermore, understanding the genetic mechanisms that determine $r_{g}$ may help in explaining the inefficiency of trait prediction across populations (Wientjes *et al.* 2015).

Following Falconer (1952), we can interpret a metric trait expressed in two populations as two different, genetically correlated traits. The additive genetic value of individual i for the trait expressed in the population that i belongs to (say, population 1) is

$$v_{i}^{P1}=h_{a,i}^{\prime }\alpha^{P1},$$

where $h_{a,i}$ is a column vector of additive genotypes (measured as allele counts, minus the mean allele count in the population) of individual i at quantitative trait loci (QTL), and $\alpha^{P1}$ is a column vector of average effects at those QTL in population 1. The additive genetic value of individual i for another population (say, population 2) is

$$v_{i}^{P2}=h_{a,i}^{\prime }\alpha^{P2},$$

where $\alpha^{P2}$ is a column vector of average effects in population 2. Conceptually, this $v_{i}^{P2}$ can be thought of as the additive genetic value for an individual in population 2 that has the same genotype as individual i. Here we define the additive genetic correlation between population 1 and population 2 ($r_{g}$) as the correlation between both additive genetic values for the individuals in population 1,

$$r_{g}=cor\left(v_{i}^{P1},v_{i}^{P2}\right)=cor\left(h_{a,i}^{\prime }\alpha^{P1},h_{a,i}^{\prime }\alpha^{P2}\right).$$ (1)

In other words, the $r_{g}$ is defined for individuals coming from population 1, which may be different from the $r_{g}$ defined for individuals coming from population 2 (See Discussion).

Equation (1) illustrates that the value of $r_{g}$ depends on the differences in average effects between populations. With non-additive effects, average effects depend on the allele frequencies in the population, and, therefore, larger differences in allele frequencies between populations are expected to result in lower values of $r_{g}$.

Note that $r_{g}$ is the correlation between the additive genetic values, not the genotypic values (*i.e.*, additive plus non-additive genetic values). In the absence of GxE-interaction, the genotypic correlation between both populations is equal to one irrespective of the presence of GxG-interactions, because the genotypic value of a genotype (*i.e.*, individual) is the same in both populations. The additive genetic correlation $\left(r_{g}\right)$ may, however, be smaller than one because the partitioning of genotypic values into additive genetic values, dominance deviations and epistatic deviations depends on the allele frequencies (Fisher 1918; Cockerham 1954; Kempthorne 1954).

A deeper understanding of the relationship between non-additive genetic effects, allele frequencies and $r_{g}$ may help geneticists to predict the value of $r_{g}$ based on the importance of dominance and epistasis in the expression of the trait, and the genetic distance between populations. Wei *et al.* (1991) studied the impact of dominance on the additive genetic correlation between a purebred and crossbred population, known as $r_{pc}$. Using a two-locus model, they showed that $r_{pc}$ indeed depends on both the magnitude of the dominance effect (d), and on the difference in allele frequencies between the populations. We are not aware of any theoretical studies that investigated the relationship between the importance of dominance and $r_{g}$ between two purebred populations.

With epistasis, $r_{g}$ is also expected to depend on the magnitude of epistatic effects and on the difference in allele frequencies between populations. Epistasis in the functional (*i.e.*, biological) sense means that the genotypic values of individuals depend on interactions between alleles or genotypes at different loci (Bateson and Mendel 1909), and there is substantial evidence for the existence of functional epistasis across species (Carlborg *et al.* 2003; Le Rouzic *et al.* 2008; Pettersson *et al.* 2011; Mackay 2015). Epistasis in the statistical sense is measured as the deviation of multi-locus genotypic values from the sum of the marginal effects (*i.e.*, average and dominance effects) of the individual loci (Fisher 1918; Cockerham 1954). Although functional epistatic interactions do not necessarily lead to substantial statistical epistasis (Cheverud and Routman 1995; Hill *et al.* 2008; Mäki-Tanila and Hill 2014), epistasis can contribute significantly to the additive genetic variance because average effects of individual loci may capture a substantial part of the functional epistasis (Hill *et al.* 2008; Mäki-Tanila and Hill 2014; Monnahan and Kelly 2015). Furthermore, epistatic variance may be ‘converted’ into additive genetic variance due to genetic drift or due to selection (Cheverud and Routman 1996; Hill 2017). Thus, epistatic interactions modify average effects of individual loci when allele frequencies change, and may therefore play an important role in the value of $r_{g}$ and its change over time.

In summary, the $r_{g}$ between populations is affected by non-additive effects in combination with differences in allele frequencies between populations. For populations in the same environment (*i.e.*, in the absence of GxE), $r_{g}$ is equal to 1 in the absence of non-additive effects or in the absence of allele frequency differences. So far, the relationship between the nature and magnitude of non-additive effects, differences in allele frequencies, and the value of $r_{g}$ remains unclear. Our objective was therefore to investigate the impact of non-additive effects on $r_{g}$ for populations that have diverged either under drift only, or under both drift and selection.

## Methods

We aimed to investigate the relationship between non-additive effects and the additive genetic correlation between populations ($r_{g}$) with small effective size, as observed in livestock. For this purpose, we simulated genotypes of quantitative trait loci (QTL) for two populations that have diverged for a number of generations under either pure drift, or under drift and selection. The populations were assumed to be kept in the same environment, so there was no GxE. We simulated traits following several scenarios that differed in the type (*i.e.*, genetic model) and the magnitude of non-additive effects (Table 1).

**Table 1:** Overview of scenarios with the genetic architecture of the trait, and their parameters for distributions of sampled dominance coefficients and epistatic coefficients

		Parameters for distributions of non-additive effects		
	Configuration	Small	Intermediate	Large
D	Dominance	$\mu_{\delta }=0.2,\sigma_{\delta }=0.30$	$\mu_{\delta }=0.2,\sigma_{\delta }=0.70$	$\mu_{\delta }=0.2,\sigma_{\delta }=1.50$
E_AA_	A*A	$\mu_{\gamma }=0.0,\sigma_{\gamma }=0.16$	$\mu_{\gamma }=0.0,\sigma_{\gamma }=0.33$	$\mu_{\gamma }=0.0,\sigma_{\gamma }=0.68$
E_DD_	D*D	$\mu_{\gamma }=0.0,\sigma_{\gamma }=0.16$	$\mu_{\gamma }=0.0,\sigma_{\gamma }=0.33$	$\mu_{\gamma }=0.0,\sigma_{\gamma }=0.68$
E_C_	Complementary	$\mu_{\gamma }=0.0,\sigma_{\gamma }=0.16$	$\mu_{\gamma }=0.0,\sigma_{\gamma }=0.33$	$\mu_{\gamma }=0.0,\sigma_{\gamma }=0.68$
E_M_	Multiplicative	$\mu_{\gamma }=0.0,\sigma_{\gamma }=0.16$	$\mu_{\gamma }=0.0,\sigma_{\gamma }=0.33$	$\mu_{\gamma }=0.0,\sigma_{\gamma }=0.68$

**Figure 1:**
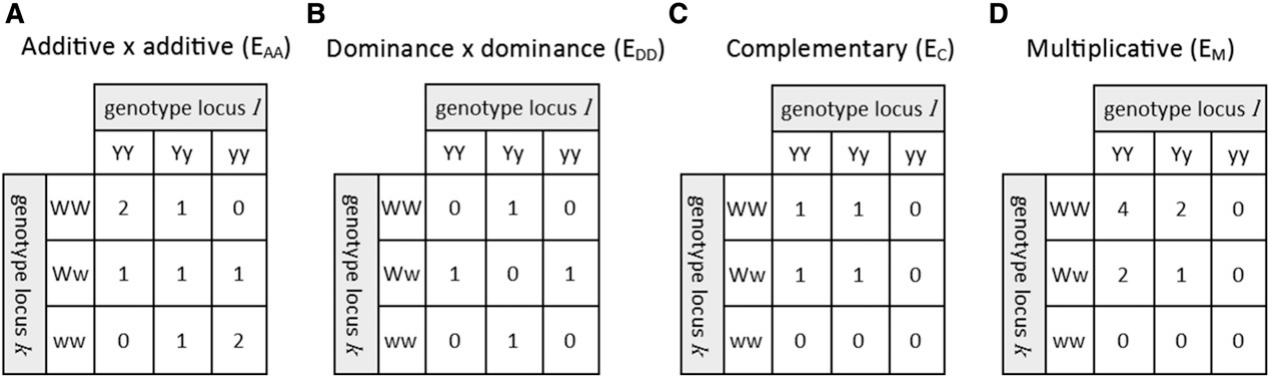
Epistatic contrasts for four biological epistatic configurations.

We considered six genetic models; a basic model with additive effects only (A), which served as a basis for comparison, and five alternative models with non-additive effects: one with only dominance effects (D), and four with only epistatic effects. With epistasis, we simulated interactions between pairs of loci that followed one of the configurations presented in Figure 1. We chose these genetic models so that there were scenarios with only dominance variance (D), scenarios with only additive by additive epistatic variance (E_AA_ and E_M_), and scenarios with all types of non-additive variance (E_C_ and E_DD_). For each genetic model, we considered three magnitudes of non-additive effects, labeled as small, intermediate, and large.

### Simulation

**Figure 2:**
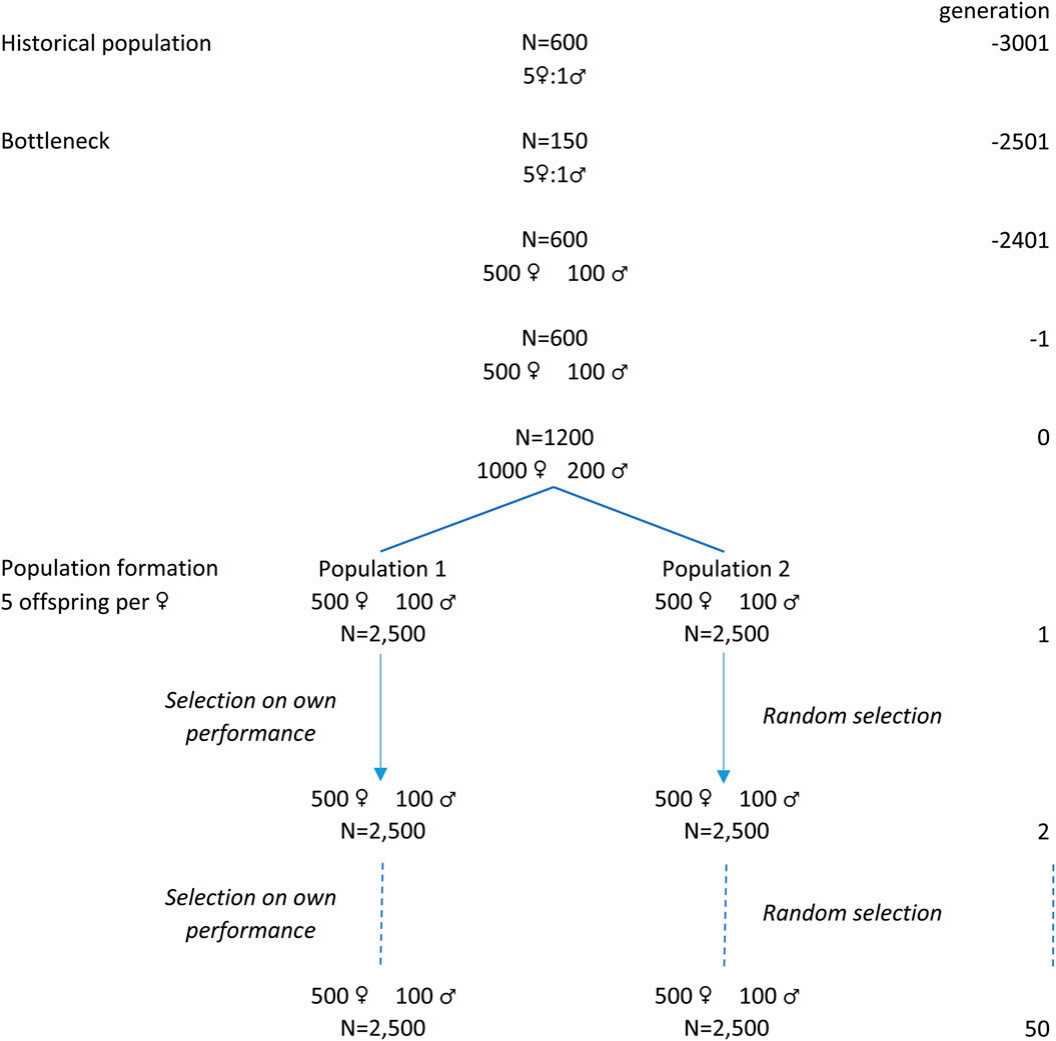
Overview of the simulated population structure.

We simulated genotypes of two livestock populations (1 and 2) that diverged for 50 generations (Figure 2). For divergence, we considered two situations: one where the populations diverged due to drift only, and one where the populations diverged also due to selection in population 1 and drift in population 2.

#### Populations:

We simulated a historical population with QMSim (Sargolzaei and Schenkel 2009) by randomly mating 100 males and 500 females starting in generation -3001. From generation -3000 to generation -2501, we simulated a bottleneck by gradually decreasing population size to 150 (25 males and 125 females) to create initial linkage disequilibrium (LD), and population size was gradually increased again to 600 during the next 100 generations. The population size remained constant from generation -2400 until -1 to allow for the development of mutation-drift equilibrium. To provide a sufficient number of individuals for the development of populations 1 and 2, we doubled the number of individuals in the last historical generation (generation 0) to 200 males and 1,000 females. This simulation resulted in an average effective population size ($N_{e}$) of ∼285 generation 0, calculated as the harmonic mean of $\frac{4N_{m}N_{f}}{N_{m}+N_{f}}$ in each preceding generation, where $N_{m}$ is the number of males and $N_{f}$ is the number of females that become parents in a generation (Falconer and Mackay 1996).

After simulating the historical population, we simulated two current populations (1 and 2). We randomly sampled 100 males and 500 females from the last historical generation to become founders of population 1. The remaining 100 males and 500 females were the founders of population 2. We will refer to the generation of founders as generation 0. Within each population, simulation continued for 50 generations by randomly mating 100 selected males with 500 selected females. Each mating resulted in 5 offspring, resulting in a total of 2,500 offspring (exactly 1,250 males and 1,250 females) in each generation. Generations were non-overlapping, meaning that in each generation, the parents were selected from the previous generation only. In the drift scenario, animals in both populations were randomly selected to become parents of the next generation. Effective population size ($N_{e}$) in the drift scenario was ∼285 in the two populations. In the selection-drift scenario, animals in population 1 were selected based on their own phenotype (mass selection), while in population 2, selection was random. In this scenario, effective population size ($N_{e}$) in population 1 was ∼250, which was calculated as 1/(2ΔF), where ΔF is the inbreeding rate estimated from the pedigree (Falconer and Mackay 1996). We simulated selection only in population 1 to reduce computation time.

#### Genome:

The simulated genome consisted of 10 chromosomes of 1 Morgan that each had 200 randomly positioned bi-allelic loci. In the first historical generation (generation -3001), we randomly sampled the allele frequencies of loci from a uniform distribution. Mutation rate was 2.5*10^−5^ during the historical generations. In generation 0, the distribution of allele frequencies had evolved to a U-shape, and we randomly selected 500 segregating loci to become QTL, which resulted in low linkage disequilibrium between QTL. There was no mutation from generation 0 to 50, because the QMSim software does not allow for mutation after the last historical generation.

#### Functional genetic effects:

Additive effects (a) of all 500 QTL were sampled from ∼N(0,1). We assumed that the size of the dominance and epistatic effects were proportional to the additive effects of the QTL involved in the interaction (Wellmann and Bennewitz 2011). We therefore sampled dominance coefficients (δ) for all QTL from $∼N(\mu_{\delta },\sigma_{\delta }^{2})$, from which dominance effects (d) were computed as δ|a|. Similarly, we sampled epistatic coefficients (γ) for all pairwise epistatic interactions from $∼N(\mu_{\gamma },\sigma_{\gamma }^{2})$, from which functional epistatic effects (ϵ) were computed as $\gamma_{kl}\sqrt{\left|a_{k}a_{l}\right|}$ where k and l denote the QTL involved in the interaction. Each QTL had an epistatic interaction with 5 randomly sampled other QTL, resulting in a total of 1250 pairwise interactions.

For both dominance and epistasis, we considered 3 magnitudes of effects: small, intermediate, and large. For all magnitudes, the mean dominance coefficient ($\mu_{\delta }$) was 0.2, and the mean epistatic coefficient ($\mu_{\gamma }$) was 0.0. The magnitude of dominance and epistatic effects were controlled by changing the standard deviation of dominance coefficients ($\sigma_{\delta }$) or epistatic coefficients ($\sigma_{\gamma }$). For dominance, $\sigma_{\delta }$ was 0.3 with small effects, 0.7 with intermediate effects, and 1.5 with large effects (Table 1). The mean and standard deviation of small dominance coefficients were chosen based on empirical results of Bennewitz and Meuwissen (2010) and Sun and Mumm (2016). For epistasis, $\sigma_{\gamma }$ was scaled such that the total functional epistatic variance was comparable to the total functional dominance variance in the scenario with the same magnitude. To this end, $\sigma_{\gamma }$ was computed as $\sqrt{(\mu_{\delta }^{2}+\sigma_{\delta }^{2})/N_{\gamma }}$, where $N_{\gamma }$ is the number of epistatic interactions per QTL, and $\mu_{\delta }^{2}$ and $\sigma_{\delta }^{2}$ are the squared mean and variance of dominance effects in the scenario with the corresponding magnitude. For example, with small epistatic effects, $\sigma_{\gamma }$ was computed as $\sqrt{(0.2^{2}+0.3^{2})/5}\approx 0.16$ (Table 1).

#### From functional dominance and epistatic effects to statistically orthogonal effects:

We simulated dominance and epistasis by introducing functional dominance and epistatic effects that are independent of allele and genotype frequencies. Our interest, however, is in statistical average, dominance and epistatic effects of QTL, which do depend on genotype frequencies (Fisher 1918; Cheverud and Routman 1995). We will describe the general procedure to obtain these statistical effects, for a general situation where there can be dominance, epistasis, or both. Note, however, that our scenarios had either dominance or epistasis, but never both. After obtaining statistical effects, we describe how we computed the additive genetic value, genotypic value and phenotype for each individual. Although genotypic values themselves are independent of genotype frequencies, the partitioning of these genotypic values into additive, dominance, and epistatic components does depend on genotype frequencies. Additive genetic values of individuals in population 1 were needed to compute $r_{g}$, and genotypic values and phenotypes were needed because selection in population 1 was based on own performance. In the following, we describe the procedure to obtain the average effects and dominance effects in population 1 ($\alpha^{P1}$). The procedure to obtain these effects in population 2 ($\alpha^{P2}$) follows naturally by replacing the genotype and allele frequencies of population 1 with the frequencies in population 2.

The procedure starts by applying the natural and orthogonal interactions (NOIA) model (Álvarez-Castro and Carlborg 2007) for each epistatic interaction between two QTL. First, functional epistatic values for the 9 possible two-locus genotypes at QTL k and l were collected in a vector $c_{kl}=t\epsilon_{kl}$, where $\epsilon_{kl}$ is a scalar representing the functional epistatic effect between QTL k and l, and t is a 9 × 1 vector of epistatic contrasts for the 9 two-locus genotypes, ordered as (WWYY, WwYY, wwYY, WWYy, ..., wwyy). The simulated epistatic contrasts in t followed one of four configurations: additive x additive (E_AA_), dominance x dominance (E_DD_), complementary (E_C_), or multiplicative (E_M_) (Figure 1). The contrasts in t were centered and scaled to a standard deviation of one, so that the contrasts were comparable between configurations. We then used genotype frequencies of QTL k and l to partition the functional epistatic values in $c_{kl}$ into 9 statistical genetic effects (Álvarez-Castro and Carlborg 2007; Vitezica *et al.* 2017)

$$b_{kl}={\left(W_{kl}^{\prime }D_{kl}W_{kl}\right)}^{-1}W_{kl}^{\prime }D_{kl}c_{kl},$$

where $D_{kl}$ is a 9x9 diagonal matrix with each of the nine genotype frequencies in the same order as in t. Matrix $W_{kl}=W_{k}⊗W_{l}$, where ⊗ denotes the Kronecker product, and $W_{k}$ and $W_{l}$ are constructed as

$$W_{x}=\left[\begin{array}{ccc} 1 & w_{a} & w_{d} \end{array}\right]=\left[\begin{array}{ccc} 1 & -(-p_{Xx}-2p_{xx}) & \frac{2p_{Xx}p_{xx}}{p_{XX}+p_{xx}-{\left(p_{XX}-p_{xx}\right)}^{2}}\\ 1 & -(1-p_{Xx}-2p_{xx}) & \frac{4p_{WW}p_{ww}}{p_{XX}+p_{xx}-{\left(p_{XX}-p_{xx}\right)}^{2}}\\ 1 & -(2-p_{Xx}-2p_{xx}) & \frac{2p_{WW}p_{Ww}}{p_{XX}+p_{xx}-{\left(p_{XX}-p_{xx}\right)}^{2}} \end{array}\right],$$ (1)

where columns relate to orthogonal contrasts for the mean (1), average effect ($w_{a}$), and dominance effect ($w_{d}$) of QTL X and where $p_{Xx}$, $p_{Xx}$, and $p_{xx}$ are the genotype frequencies of QTL X. The resulting vector of statistical genetic effects is

$$b_{kl}={\left[\mu ,\alpha_{kl}^{k},d_{kl}^{k},\alpha_{kl}^{l},{\left(\alpha \alpha \right)}_{kl},{\left(d\alpha \right)}_{kl},d_{kl}^{l},{\left(\alpha d\right)}_{kl},{\left(dd\right)}_{kl}\right]}^{\prime },$$ (2)

where $\alpha_{kl}^{k}$ and $\alpha_{kl}^{l}$ are the terms that contribute to average effects of QTL k and l. The other terms in $b_{kl}$ contribute to dominance effects ($d_{kl}^{k},d_{kl}^{l}$) of individual QTL and to epistatic effects of interacting QTL (${\left(\alpha \alpha \right)}_{kl},{\left(d\alpha \right)}_{kl},{\left(\alpha d\right)}_{kl},{\left(dd\right)}_{kl}$).

We repeated this procedure of partitioning functional epistatic effects into statistical genetic effects for all pairwise interactions between QTL. Each QTL was involved in 5 epistatic interactions and therefore has 5 terms that contribute to its average effect. Following this reasoning, the average effect of QTL k in population 1 with epistasis is

$$\alpha_{k}^{P1}=a_{k}+\left(1-2p_{k}^{P1}\right)d_{k}+\overset{N_{\gamma }}{\underset{l\in \mathbb{Z}}{\sum }}\alpha_{kl}^{k},$$

where $p_{k}^{P1}$ is the frequency of the counted allele of QTL k in population 1, ℤ is the set of loci that QTL k interacts with, and $N_{\gamma }=5$. Note the difference between “additive effect” (a) and “average effect” (α); the additive effect a is half the difference in genotypic value between both opposing homozygotes, whereas the average effect (α) is the (statistical) marginal effect of the QTL. Throughout this manuscript, we will use the term “functional additive effect” to refer to a, and “average effect” (*i.e.*, statistical substitution effect) to refer to α.

In our simulations, we needed to compute phenotypes of selection candidates in each generation, for which we needed the statistical dominance effect ($d^{*}$) of each QTL as well. The dominance effect of QTL k in population 1 with epistasis is

$$d_{k}^{P1}{}^{*}=d_{k}+\overset{N_{\gamma }}{\underset{l\in \mathbb{Z}}{\sum }}d_{kl}^{k}$$

#### Additive genetic values and phenotypes:

We computed additive genetic values (v) of selection candidates in population 1 for the trait expressed in both population 1 and 2. Their genotypic values (g) and phenotypes were only computed for the trait expressed in population 1. The additive genetic value of individual i for the trait expressed in population 1 (2) were computed as $v_{i}^{P1}=h_{a,i}^{\prime }\alpha^{P1}$ ($v_{i}^{P2}=h_{a,i}^{\prime }\alpha^{P2}$), and genotypic values for the trait expressed in population 1 were computed as

$$g_{i}^{P1}=v_{i}^{P1}+h_{d,i}^{\prime }d^{P1}{}^{*}+h_{a,i}^{\prime }⊗h_{a,i}^{\prime }{\left(\alpha \alpha \right)}^{P1}+h_{a,i}^{\prime }⊗h_{d,i}^{\prime }{\left(\alpha d\right)}^{P1}+h_{d,i}^{\prime }⊗h_{a,i}^{\prime }{\left(d\alpha \right)}^{P1}+h_{d,i}^{\prime }⊗h_{d,i}^{\prime }{\left(dd\right)}^{P1},$$

where $h_{a,i}$ is a column vector of additive genotype indicators for individual i, and $h_{d,i}$ is column a vector of dominance genotype indicators for individual i. These indicators were coded following the NOIA parameterization as denoted in the rows of $w_{a}$ and $w_{d}$ (Equation 1) for genotypes XX, Xx, and xx, respectively. Phenotypes with a broad sense heritability of 0.5 were computed as $y^{P1}=g^{P1}+e^{P1}$, where $e^{P1}∼N(0,\sigma_{e}^{2})$, and $\sigma_{e}^{2}$ was equal to the variance of genotypic values ($\sigma_{g}^{2}$).

### Computing parameters of interest

The parameters of interest were (1) the genetic correlation between the trait in population 1 and the trait in population 2 ($r_{g}$), and (2) the average absolute difference in allele frequencies between populations ($\bar{\text{Δ}p}$). For each generation, we computed $r_{g}$ as the Pearson correlation between the additive genetic values of individuals in population 1 for the trait expressed in the two populations (equation (1)). Effectively, this $r_{g}$ is a weighted correlation between $\alpha^{P1}$ and $\alpha^{P2}$, where the weights depend on the allele frequencies in population 1. Hence, the $r_{g}$ computed as the correlation of additive genetic values of individuals in population 2 may give different results because the genotypes sampled from population 2 result in different weights than those sampled from population 1 (see Discussion). For each generation, we computed $\bar{\text{Δ}p}$ as $\sum^{\text{​}}(|p_{k}^{A}-p_{k}^{B}|)/500$. We chose this parameter as a measure for population divergence, because we expect that there is a linear relationship between $\bar{\text{Δ}p}$ and $r_{g}$. These parameters were computed for generation 1 to 5, and for every 5^th^ generation after generation 5, to limit computation time.

### Replicates

We ran the simulation with drift 50 times, resulting in 50 sets of genotypes (*i.e.*, replicates). For each of those replicates, we computed $\bar{\text{Δ}p}$ and $r_{g}$ for each of the scenarios (*i.e.*, genetic model and magnitude). We ran the simulations with both selection and drift for each scenario separately, because the selection of parents in population 1 depended on the genetic model. To limit computation time, we used 20 replicates for each scenario with selection.

### Data availability

The data used in this study can be reproduced with the files and seeds in the following GitHub repository: https://git.wageningenur.nl/duenk002/rg-and-non-additive-effects. Supplemental material available at figshare: https://doi.org/10.25387/g3.10252856.

## Results

First, for each scenario with selection, we show the change in mean genotypic value ($\bar{g}$) and the change of additive genetic variance (V_A_) in population 1 across generations, to illustrate how population 1 evolved over time. Second, we report realized fractions of additive, dominance and epistatic variance in generation 1 and 50. Third, for scenarios with small non-additive effects, we show the effects of the genetic model and of applying selection on the additive genetic correlation ($r_{g}$) and the difference in allele frequency ($\bar{\text{Δ}p}$) between populations. Fourth, for each genetic model with selection, we investigate the impact of the magnitude of non-additive effects and the number of generations since divergence. Finally, we investigate the relationship between $r_{g}$ and $\bar{\text{Δ}p}$ across genetic models and within genetic models. All results presented refer to generation 50 and to scenarios with small non-additive effects, unless otherwise stated.

### Mean genotypic value and variance components

With all scenarios, the mean genotypic value expressed in genetic standard deviations ($\bar{g}$) in population 1 increased due to selection (Figure S 1). With all genetic models, the increase in $\bar{g}$ was smaller when the magnitude of non-additive effects was larger. This result was expected, because the marginal effects of alleles may change over time in the presence of non-additive effects, reducing the effectiveness of selection. The increase in $\bar{g}$ was largest with model A, and it was smallest with model E_DD_ and large non-additive effects. There were only small differences in $\bar{g}$ between models D, E_AA_, E_C_, and E_M_.

The additive genetic variance in population 1 (V_A_) decreased due to selection with all scenarios (Table 2 and Figure S 2). With genetic model A, E_AA_, E_C_ and E_M_, about 95–98% of V_A_ was lost after 50 generations of selection, whereas with D and E_DD_, 88–95% of V_A_ was lost. A change in magnitude of non-additive effects did not substantially affect the decrease in V_A_, except with genetic models D and E_DD_, where more additive genetic variance was preserved with larger non-additive effects. In the drift scenario, the average loss of V_A_ was about 7% for all scenarios (results not shown).

**Table 2:** Fractions of additive (V_A_), dominance (V_D_), and epistatic (V_I_) variances with respect to the total genetic variance in generation 1 and generation 50 with selection. Reported values are averages of 20 replicates

		Generation 1			Generation 50		
Scenario	Effect size	V_A_	V_D_	V_I_	V_A_	V_D_	V_I_
D	Small	0.961	0.039	0.000	0.924	0.076	0.000
	Intermediate	0.871	0.129	0.000	0.511	0.489	0.000
	Large	0.702	0.298	0.000	0.198	0.802	0.000
E_AA_	Small	0.992	0.000	0.008	0.997	0.000	0.003
	Intermediate	0.976	0.000	0.024	0.988	0.000	0.012
	Large	0.952	0.000	0.048	0.969	0.000	0.031
E_DD_	Small	0.910	0.064	0.026	0.703	0.289	0.008
	Intermediate	0.751	0.173	0.076	0.358	0.602	0.040
	Large	0.528	0.333	0.139	0.146	0.752	0.101
E_C_	Small	0.985	0.012	0.003	0.947	0.051	0.001
	Intermediate	0.947	0.044	0.009	0.737	0.250	0.013
	Large	0.871	0.105	0.024	0.471	0.511	0.017
E_M_	Small	0.998	0.000	0.002	0.999	0.000	0.001
	Intermediate	0.993	0.000	0.007	0.995	0.000	0.005
	Large	0.983	0.000	0.017	0.989	0.000	0.011

In generation 1, scenarios that had only additive genetic (V_A_) and epistatic variance (V_I_), V_A_ accounted for the largest, and V_I_ for the smallest fraction of the total genetic variation (Table 2). The largest fraction of V_I_ was realized with genetic model E_AA_ (max. 0.048), followed by E_DD_ (max. 0.033), E_C_ (max. 0.024) and E_M_ (max. 0.017). The largest fraction of dominance variance (V_D_) was realized with model E_DD_ (max. 0.364), followed by D (max. 0.298) and E_C_ (max. 0.105). With genetic models D, E_DD_ and E_C_, the fraction V_D_ increased and V_A_ decreased across generations, especially with intermediate or large effects (Table 2, generation 50). The fraction V_I_ remained relatively constant across generations with all scenarios.

### Effect of genetic model and of selection on $r_{g}$

**Figure 3:**
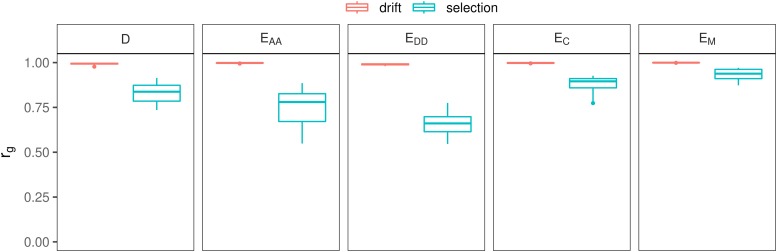
Effect of genetic model on $r_{g}$ with small non-additive effects, under drift only, or under drift and selection.

For all genetic models and small non-additive effects, $r_{g}$ was lower with selection than with drift only (Figure 3). With drift only, $r_{g}$ was between 0.99 and 1 for all genetic models. After 50 generations of selection, average $r_{g}$ was lowest with genetic model E_DD_ (0.65), followed by E_AA_ (0.75), D (0.83), E_C_ (0.83) and finally E_M_ (0.94). There was a tendency that scenarios with the largest non-additive variance in generation 1 had the smallest $r_{g}$ in generation 50 (Figure S 3). Note that the $r_{g}$ was always equal to 1 with the additive model (A) (results not shown).

**Figure 4:**
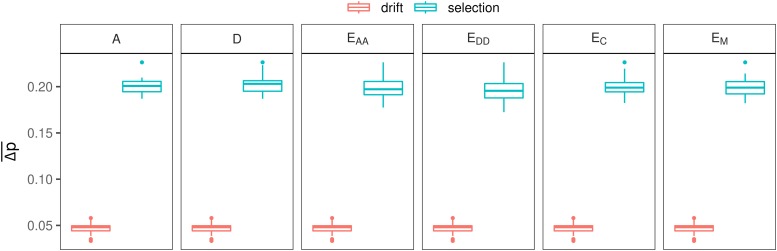
Effect of genetic model on the difference in allele frequencies between populations, under drift only, or under drift and selection.

As expected, $\bar{\text{Δ}p}$ was larger with selection than with drift, and was the same across all genetic models with drift (0.05; Figure 4). With selection, $\bar{\text{Δ}p}$ with non-additive models was very similar (around 0.20) to the value with an additive model.

### Effect of the magnitude of non-additive effects

**Figure 5:**
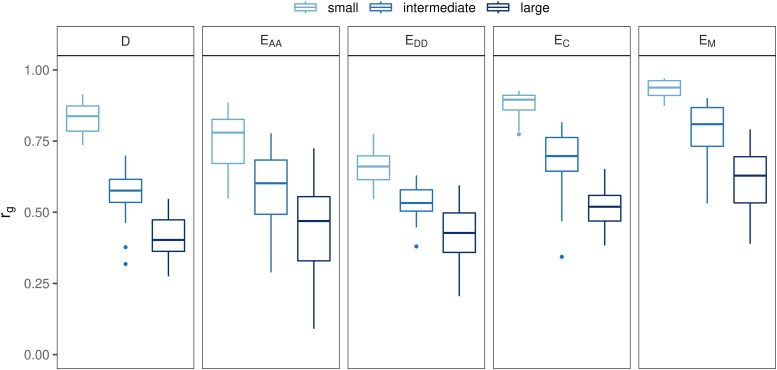
Effect of magnitude of non-additive effects on $r_{g}$ for all genetic models, where population 1 was selected and population 2 was not selected.

For all genetic models and with selection, $r_{g}$ decreased with increasing magnitude of non-additive effects (Figure 5). With genetic model D, $r_{g}$ dropped about 31% from small to intermediate, and about 27% from intermediate to large dominance effects. With all epistatic models, the drop in $r_{g}$ with increasing magnitude was smaller (16–23%) than with D.

**Figure 6:**
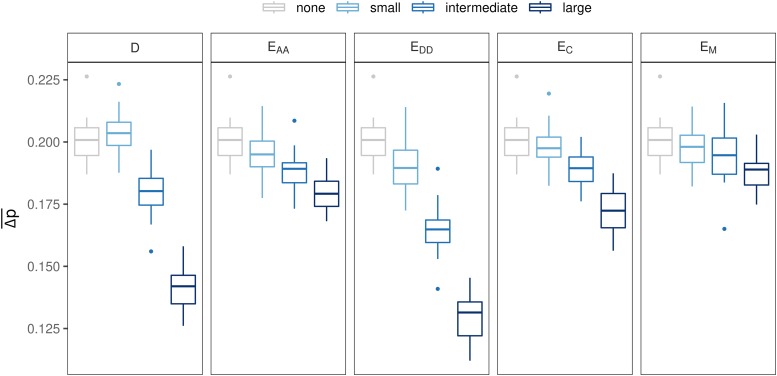
Effect of magnitude of non-additive effects on the difference in allele frequencies between populations under selection. In each subplot, the additive scenario (A) was included for reference (*i.e.*, magnitude “none”).

For all genetic models with selection, the average absolute difference in allele frequency between lines ($\bar{\text{Δ}p}$) decreased with increasing magnitude of non-additive effects, especially with D and E_DD_ (Figure 6). With model D, $\bar{\text{Δ}p}$ was 0.18 with intermediate dominance effects, and 0.141 with large effects. With E_DD_, $\bar{\text{Δ}p}$ was 0.162 with intermediate epistatic effects, and 0.130 with large effects. With the other epistatic models (E_AA_, E_C_ and E_M_), the effect of an increase in magnitude on $\bar{\text{Δ}p}$ was much smaller (∼0.19 with intermediate and ∼0.18 with large effects).

### Effect of number of generations since divergence

**Figure 7:**
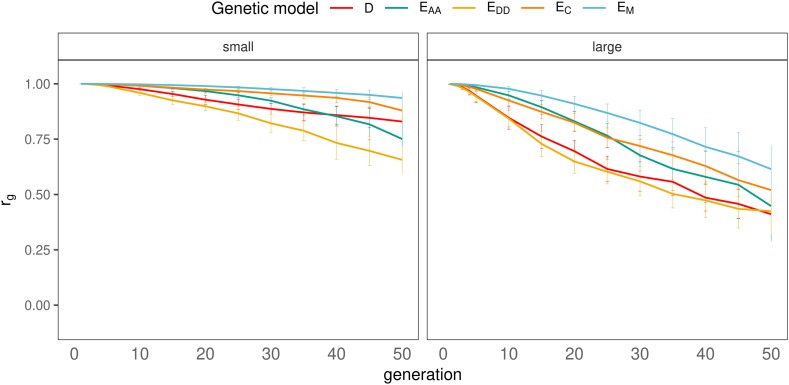
Effect of number of generations since divergence on $r_{g}$ for all genetic models with small (left) or large (right) non-additive effects.

With all scenarios, $r_{g}$ decreased with the number of generations since divergence, and the rate of decrease was relatively small during the first five generations ($r_{g}>0.94$), especially when the non-additive effects were small ($r_{g}>0.98$) (Figure 7). After the first five generations, the rate of decrease in $r_{g}$ differed across genetic models. There was a considerable difference between genetic models, the E_M_ model showed the smallest decline of $r_{g}$ over time, and the E_DD_ model showed the largest decline. With large non-additive effects, models E_M_ and E_AA_ tended to show an accelerated decrease in $r_{g}$ across generations, whereas models D, E_C_ and E_DD_ tended to show a decelerated decrease in $r_{g}$ (Figure 7).

**Figure 8:**
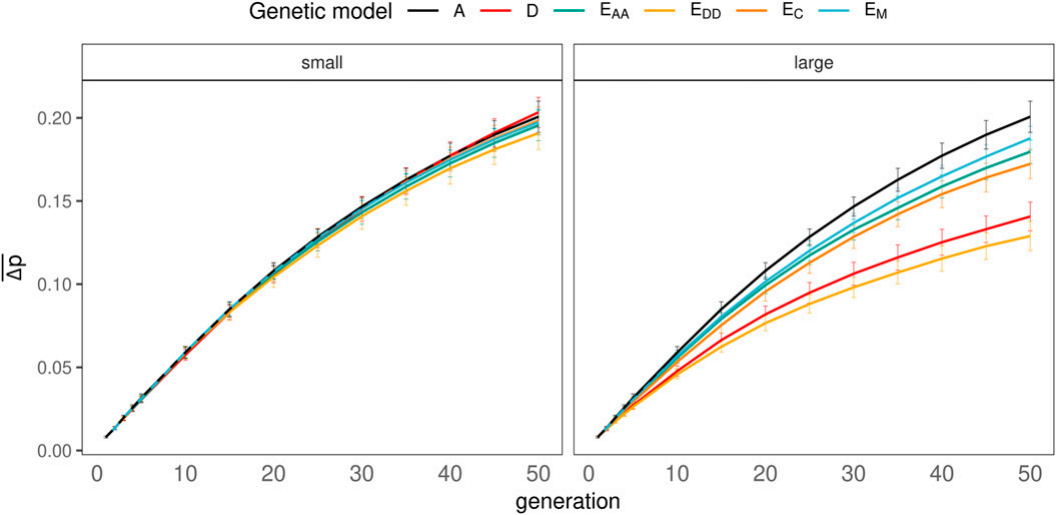
Effect of number of generations since divergence on the difference in allele frequencies between populations, for all genetic models and small (left) or large (right) non-additive effects. Dotted line represents additive trait.

With all scenarios, the average absolute difference in allele frequency between lines ($\bar{\text{Δ}p}$) increased with the number of generations since divergence (Figure 8). In contrast to the result of the genetic correlation with small non-additive effects (Figure 7), $\bar{\text{Δ}p}$ was remarkably similar between the genetic models (Figure 8). With large effects, models D and E_DD_ showed a smaller $\bar{\text{Δ}p}$ than models E_M_, E_AA_, and E_C_.

In summary, for each genetic model, $r_{g}$ was smallest with selection, large non-additive effects and many generations since divergence. Overall, the smallest realized value of $r_{g}$ after 50 generations of divergence was achieved with genetic model D or E_DD_ ($r_{g}\approx 0.41$ for both).

### Relationship Between $r_{g}$ and $\bar{\Delta p}$

**Figure 9:**
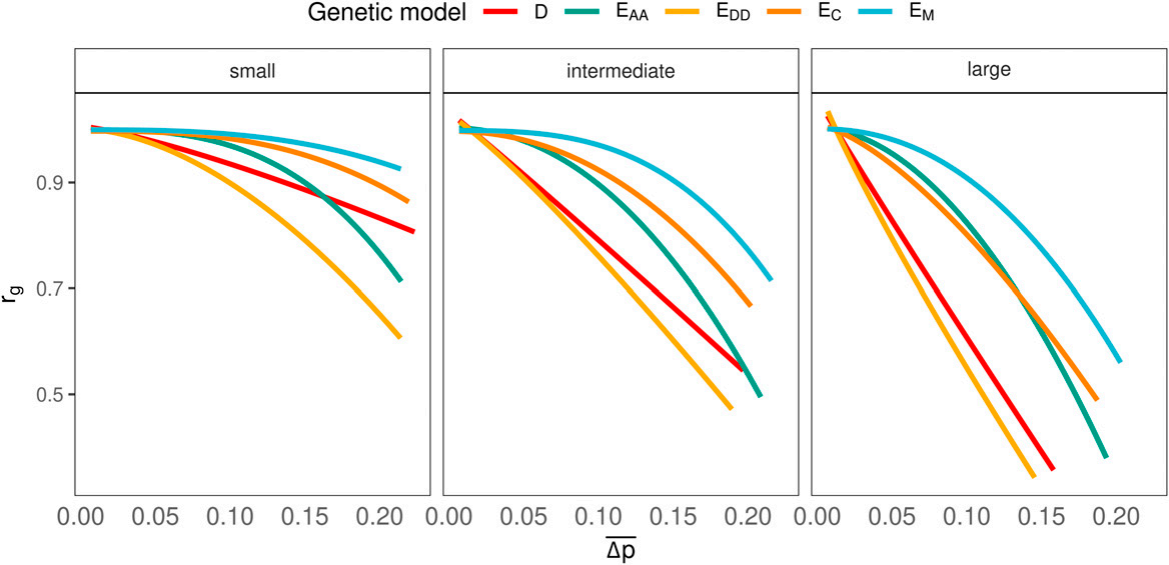
Relationship between $r_{g}$ and the difference in allele frequencies between populations for all scenarios.

For all genetic models, there was a clear negative relationship between $\bar{\text{Δ}p}$ and $r_{g}$ (Figure 9), and the relationship was strongest for genetic models showing the strongest decline of $r_{g}$ with time (Figure 7). This result suggests that differences between genetic models in the decline of $r_{g}$ over time originate from different impacts of $\bar{\text{Δ}p}$ on $r_{g}$, and not from differences in $\bar{\text{Δ}p}$ *per se*. For example, with small non-additive effects and after 50 generations of divergence, the value of $r_{g}$ was different between genetic models, whereas the realized $\bar{\text{Δ}p}$ was very similar (Figure 9). In other words, $r_{g}$ is a function of $\bar{\text{Δ}p}$ and of genetic architecture.

## Discussion

Our objective was to investigate the relationship between non-additive effects, differences in allele frequencies between populations ($\bar{\text{Δ}p}$), and the genetic correlation between populations ($r_{g})$. We simulated genotype data of two populations that have diverged for a number of generations under drift only, or drift and selection, and we simulated traits where the genetic model and magnitude of non-additive effects were varied.

We computed $r_{g}$ as the correlation between additive genetic values of individuals in population 1, for the trait expressed in population 1 and 2. Effectively, this $r_{g}$ is a weighted correlation between average effects in population 1 ($\alpha^{1}$) and 2 ($\alpha^{2}$), where the weights depend on the sample of genotypes that were used to compute the additive genetic values. This suggests that different values of $r_{g}$ could have been obtained when using the additive genetic values of individuals in population 2, because of differences in genotype frequencies between populations. We chose, however, to focus on population 1 because we were also interested in the change of allele frequencies over time due to selection. This approach leads to values of $r_{g}$ that indicate whether information from an unselected population (population 2) can be used to predict additive genetic values in a selected population (population 1).

### Realized variance components

Because little is known about the quantity and magnitude of dominance and epistatic effects in reality, we considered a range of functional non-additive effect sizes and epistatic configurations. Realized proportions in our simulations (Table 2) did not always match with those observed in real data. For example, with large dominance effects, the fraction of dominance variance was 30%, which is uncommon in real data (Ertl *et al.* 2014; Lopes *et al.* 2016; Moghaddar and van der Werf 2017; Joshi *et al.* 2018). Similarly, scenario E_DD_ also resulted in more dominance variance than expected in real populations, especially with large epistatic effects (33%). Empirical studies on livestock (Bennewitz and Meuwissen 2010) and crops (Sun and Mumm 2016) found that approximately 0.3% of loci show overdominance, which is comparable to our scenario with small dominance effects (0.5% overdominance). Furthermore, the scenario with small dominance effects resulted in a small proportion of dominance variance, and might therefore be most realistic for actual populations.

In contrast to our realized proportions of dominance variance, proportions of epistatic variance were lower (max. 5%) than estimates from an empirical study on litter size in pigs (about 26%) (Vitezica *et al.* 2018), though the standard error of that estimate was large (about 22%). Further evidence of statistical epistatic effects is scarce, probably because methods used for the detection of statistical epistasis are frequently underpowered (Wei *et al.* 2014). Furthermore, it has been suggested that incomplete LD between genomic markers and QTL may create the illusion of epistasis, making inference about the importance of epistasis from genome-wide regression studies difficult (Wei *et al.* 2014; Zan *et al.* 2018; de los Campos *et al.* 2019). In contrast to the lack of evidence of statistical epistasis, there is substantial evidence that physiological epistasis is abundant in several classes of organisms (Carlborg *et al.* 2003; Le Rouzic *et al.* 2008; Pettersson *et al.* 2011; Mackay 2015). Nevertheless, large epistatic effects between pairs of loci are believed to be unlikely (Wei *et al.* 2014), and the contribution of epistatic variance to the total genetic variance is expected to be small (Hill *et al.* 2008).

In summary, among the scenario’s we studied here, scenarios D and E_DD_ with small effects, and scenarios E_AA_, E_C_ and E_M_ are probably most realistic, because these scenarios always resulted in little dominance (max. 7%) and epistatic (max. 5%) variance.

### Effect of genetic model on $r_{g}$

For the dominance model (D), we observed that $r_{g}$ decreased with increasing size of dominance effects and with increasing difference of allele frequencies between populations. In some cases, the $r_{g}$ can be negative due to dominance alone, as shown for a two-locus model (Wei *et al.* 1991). Such low values of $r_{g}$ were, however, only obtained with scenarios where both loci showed substantial overdominance, and where the difference in allele frequencies between the two populations was at least 0.3 for one of the loci. In our study, we considered many loci and the distributions of dominance effects was based on empirical results (Bennewitz and Meuwissen 2010; Sun and Mumm 2016). These distributions resulted in only a fraction of loci showing overdominance (*i.e.*, 0.5% for small effects, 16% for intermediate effects, and 51% for large effects). Furthermore, our simulations resulted in U-shaped distributions of allele frequencies in the last generation of the historical population, which agrees with expectations based on neutral theory (Kimura and Crow 1964; Goddard 2001). After the two populations separated, allele frequency differences between populations were a result of drift and/or selection. We therefore believe that our simulations represent a more realistic model of quantitative traits and population divergence than those in Wei *et al.* (1991). In conclusion, given that dominance variance is usually small and overdominance does not occur frequently, our results show that it is unlikely that true $r_{g}$ values lower than 0.80 are due to dominance effects alone.

In another simulation study, where the fraction of loci showing overdominance was 12%, realized $r_{g}$ was 0.78 (Esfandyari *et al.* 2015). Although the fraction of loci showing overdominance in that study was comparable to our scenario with intermediate dominance effects, our realized $r_{g}$ in that scenario was much lower (0.57). This difference is likely due to the smaller number of generations that populations diverged in the study of Esfandyari *et al.* (2015).

With epistasis, $r_{g}$ decreased with increasing size of epistatic effects and with increasing difference of allele frequencies between populations, and the value of $r_{g}$ depended on the nature of the epistatic interaction (*i.e.*, configuration). In addition, there was a tendency for configurations that resulted in large initial non-additive variance to result in smaller values of $r_{g}$ (Figure S 3). Even though large epistatic effects are unlikely and epistatic variance is expected to be small, $r_{g}$ could be as low as 0.45 for supposedly realistic epistatic scenarios.

To our knowledge, the relationship between the nature of epistasis and $r_{g}$ has not been studied before. The mechanism behind differences in $r_{g}$ between epistatic models can be illustrated with an example of two interacting loci. Suppose that both loci have an additive effect (a) of 1, an epistatic coefficient (γ) of 0.5, and the allele frequency at locus 1 ($p_{1}$) is the same in both populations (here we use 0.10). Then, we study the effect of allele frequency difference between populations at locus 2 ($\text{Δ}p_{2}$) on the difference in average effects between populations (Δα) for locus 1 and 2. Results show that E_AA_ and E_M_ interactions only affect the α of the locus with fixed p (locus 1), whereas E_DD_ and E_C_ interactions affect the α at both loci (Figure S 4). Note that this result was the same with different values for a, γ, or $p_{1}$. This shows that, in general, E_AA_ and E_M_ interactions create a dependency of α at a locus on the allele frequency of all loci it interacts with, whereas E_DD_ and E_C_ interactions also create a dependency of α on the allele frequency of the locus itself. These mechanisms may contribute to the differences in $r_{g}$ between genetic models, because the interplay between differences in allele frequencies and $r_{g}$ depends on the genetic model.

### Effect of magnitude of non-additive effects on $r_{g}$

As expected, an increase in magnitude of dominance effects resulted in a lower $r_{g}$, which is in line with results from Wei *et al.* (1991). Similarly, an increase in magnitude of epistatic effects also resulted in a lower $r_{g}$. An important question is whether this decrease of $r_{g}$ due to an increase in magnitude continues until the theoretical limit of $r_{g}=-1$ is reached. Additional analyses revealed that $r_{g}$ appears to asymptote with increasing magnitude of non-additive effects. In these analyses, we repeated our original simulations of genetic models D and E_AA_, using non-additive effects that were multiplied by 100 for all magnitudes. Results from those simulations showed that the difference in $r_{g}$ between “small”, “intermediate”, or “large” effects had indeed disappeared (Figure S 5), and that the lower bound of realized values for $r_{g}$ was ∼0.25 with scenario D and ∼0.36 with scenario E_AA_.

To show the mechanism behind this result, we again consider a two-locus model where, like before, both loci have an additive effect (a) of 1, the allele frequency of locus 1 ($p_{1}$) is 0.10 in both populations and $\text{Δ}p_{2}=0.20$. We studied the effect of the magnitude of the epistatic effect (γ) on the absolute difference in average effects between populations, relative to the absolute value of α in population 1 ($\text{Δ}\alpha /\alpha_{A}$). We observed that for all epistatic models, especially for larger values of γ, both Δα and $\alpha_{A}$ increase roughly linearly with γ, and that therefore $\text{Δ}\alpha /\alpha_{A}$ stops increasing with large values of γ (Figure S 6). Note that the same mechanism was observed with dominance when $p_{2}$ was the same in both populations and $\text{Δ}p_{1}=0.20$. Hence, a change in magnitude equally affects the variance of α’s in the two populations, and the covariance between them. As a result, $r_{g}$ is unaffected by a change in size of non-additive effects when non-additive effects are already large. In conclusion, when non-additive effects are very large, $r_{g}$ no longer depends on the magnitude of non-additive effects relative to the magnitude of functional additive effects. At that point, there is a lower bound of $r_{g}$ that is determined by the nature of the non-additive effects (*i.e.*, type of inter-allelic interaction) and by the difference in allele frequencies between populations.

### Number of epistatic interactions

In the epistatic scenarios, we assumed that each locus interacted with 5 other loci. Because little is known about the number of interactions per locus ($N_{\gamma }$) in reality, we tested whether our results were sensitive to a change in $N_{\gamma }$. For that purpose, we repeated all simulations of epistatic scenarios with $N_{\gamma }=100$. Note that the total functional epistatic variance with $N_{\gamma }=100$ was the same as with $N_{\gamma }=5$, because the epistatic coefficients were scaled with $N_{\gamma }$, so that the product $N_{\gamma }\sigma_{\gamma }^{2}$ is constant. This analyses resulted in values of $r_{g}$ that were very similar to those of our original simulations (results not shown), suggesting that, in our simulations, the value of $r_{g}$ depends on the level of total functional epistatic variance, which scales similarly with $N_{\gamma }$ or $\sigma_{\gamma }^{2}$.

### Effect of selection on $r_{g}$

Non-additive effects and selection create a complex interplay between average effects, the difference in allele frequencies between populations ($\bar{\text{Δ}p}$) over time, and their effects on $r_{g}$. For a trait with small dominance effects under selection, we observed that $\bar{\text{Δ}p}$ was almost the same as for an additive trait (Figure 8). We expected, however, that directional dominance would reduce $\bar{\text{Δ}p}$, because the average effect at a locus can become smaller or even switch sign when the frequency of the favorable dominant allele increases (Falconer and Mackay 1996). This change in average effects would affect the change in allele frequencies over time due to selection in population 1, because the selection pressure at loci may change. A reduction in $\bar{\text{Δ}p}$ with small dominance effects was not observed, probably because only a small fraction of loci showed full- or over-dominance. Indeed, with large dominance coefficients (so that the fraction of loci showing over-dominance was much larger compared to with small dominance coefficients) $\bar{\text{Δ}p}$ was smaller (Figure 6). In real data, however, we do not expect a large fraction of loci that show full- or over-dominance (Wellmann and Bennewitz 2011). It is therefore unlikely that dominance significantly affects the change in allele frequencies over time due to selection, compared to a purely additive trait.

For a trait with epistatic effects under selection, we observed that $\bar{\text{Δ}p}$ was a bit smaller than that for a trait with only additive effects (Figure 8). Similar to the models with only dominance effects, this reduction in $\bar{\text{Δ}p}$ was expected because the average effect at a locus can become smaller or switch sign over time in the presence of epistasis. How epistasis affects the change in allele frequencies due to selection depends on the directionality of the epistatic interaction effect. Theory suggests that, compared to pure additivity, positive interactions (*i.e.*, in the same direction as the additive effects) will promote the selection of favorable alleles, whereas negative interactions (*i.e.*, in the opposite direction from the additive effects) will suppress the selection of favorable alleles (Carter *et al.* 2005; Hansen 2013; Paixão and Barton 2016). We chose to simulate both positive and negative interactions with equal probabilities, because empirical studies suggest that epistatic interactions are not biased in being either positive or negative (*i.e.*, they are non-directional) (Mackay 2014). Our results showed that, for a trait with intermediate epistatic effects, the net effect of having both positive and negative interactions was a decrease in fixation rate of favorable alleles (*i.e.*, with a positive a), and an increase in fixation rate of unfavorable alleles (*i.e.*, with a negative a) compared to an additive trait (Figure S 7). Similar results were found by Esfandyari *et al.* (2017). In conclusion, epistatic effects may affect $r_{g}$ through two related mechanisms. First, with an epistatic model and when selection takes place in one of the populations, the difference in allele frequencies between populations may be smaller compared to an additive model. This reduction occurs because negative interactions decrease the fixation rates of favorable alleles, and increase those of unfavorable alleles. Second, for given allele frequency differences, the value of $r_{g}$ depends on the nature of the epistatic interaction.

### Loss of additive genetic variance

Selection experiments in Drosophila, maize, and *Escherichia coli* have shown that additive genetic variation (V_A_) can be maintained for at least 100 generations (Hill 2016). Some researchers suggested that this preservation of V_A_ may be due to the conversion of non-additive genetic variance to additive genetic variance (Cheverud and Routman 1996; Hallander and Waldmann 2007; Hill 2017). Simulation studies, however, have failed to show a preservation of V_A_ due to this conversion (Carter *et al.* 2005; Esfandyari *et al.* 2017). Similarly, our simulations showed little conversion of non-additive genetic variance to V_A_ with genetic models E_AA_ and E_M_, and no conversion with other genetic models (Table 2). As a result, almost all additive genetic variance was lost after 50 generations (Figure S 2).

The large loss of additive genetic variance in our simulations may be due to two reasons. First, there was little epistatic variance in generation one that could be ‘converted’ to V_A_ in subsequent generations (Hill *et al.* 2008; Mäki-Tanila and Hill 2014). This was largely because the allele frequency distribution was strongly U-shaped in generation one. Second, mutational variance was zero because there were no mutations simulated after the historical generation. Even though these mechanisms may explain some of the loss of $V_{A}$ in our simulations, the issue still remains that, to date, simulations have failed to convincingly reproduce the conservation of $V_{A}$ observed in reality (Johnson and Barton 2005; Walsh and Lynch 2018).

### Practical relevance

In our simulations, there was selection in only one of the populations, while the other population was unselected. In reality, populations may have been divergently selected (*e.g.*, Friesian Holstein *vs.* Angus cattle), resulting in larger differences in allele frequencies than simulated here. Hence, $r_{g}$ between divergently selected populations may be smaller than observed in our simulations.

In this study, we assumed that there were no genotype x environment interactions (GxE), so that $r_{g}$ values smaller than one were only due to non-additive effects. In reality, both non-additive effects and GxE may contribute to $r_{g}$ values being smaller than one. The relative importance of non-additive effects and GxE can be inferred from the difference between estimated $r_{g}$, from a design where the populations were tested in different environments, and from a design where one of the populations was tested in the environment of the other population. This approach is similar to what was proposed by Wientjes and Calus (2017) to dissect the components of the genetic correlation between purebred and crossbred performance. However, to our knowledge, there are no studies that have used this approach to disentangle the effects of non-additive effects and GxE on $r_{g}$. This study shows that, even without GxE, the $r_{g}$ can be substantially smaller than one, and sometimes even close to zero.

Estimated genetic correlations between two populations (${\hat{r}}_{g}$) may differ across traits (*e.g.*, Lund *et al.* 2011; Karoui *et al.* 2012; Porto-Neto *et al.* 2015). For example, in dairy cattle, ${\hat{r}}_{g}$ of fertility traits tended to be lower than those of fat yield and milk production (Karoui *et al.* 2012). The results from the present study suggest that such differences in ${\hat{r}}_{g}$ may indicate differences in the underlying genetic model between traits (*i.e.*, in the importance of non-additive effects). Although this may be the case, differences in ${\hat{r}}_{g}$ between traits can arise through other mechanisms as well. First, ${\hat{r}}_{g}$ often include a component due to GxE interactions. Such GxE interactions may be more important for some traits than for others, resulting in differences in ${\hat{r}}_{g}$ between traits. Second, different traits are influenced by (at least partly) different QTL, and some traits may have been under stronger selection than others. As a result, the differences in allele frequencies at QTL between populations may vary across traits. These mechanisms may result in differences in ${\hat{r}}_{g}$ between traits, even when the underlying genetic models of those traits are similar. It is therefore questionable whether inferences can be made about differences in genetic model among traits, based on differences in ${\hat{r}}_{g}$.

The results in this study may be relevant for the prediction of additive genetic values across populations using genomic information. In this strategy, termed across-population genomic prediction, average effects at markers are estimated in one population, and used to compute additive genetic values in another population (de Roos *et al.* 2009; Hayes *et al.* 2009). It has been suggested that the inefficiency of across-population genomic prediction is partly due to differences in linkage disequilibrium between markers and QTL. This insight has inspired the use of whole-genome sequence (WGS) data, because in WGS data, genotypes of the QTL themselves are included (Iheshiulor *et al.* 2016; Raymond *et al.* 2018a; Raymond *et al.* 2018b). The results of the current study suggest, however, that even when QTL genotypes are known and their average effects are accurately estimated in one population, across-population genomic prediction may be inefficient, because $r_{g}$ can differ considerably from one, even when genetic variance is mostly additive. This view is supported by the results of Raymond *et al.* (2018b), who reported that although the $r_{g}$ estimated from putative QTL was higher than the estimate from regular marker data, it was still lower than one.

Similar to across populations, genomic prediction from current to future generations may be inefficient because of changes in allele frequencies, and the subsequent changes in average effects at QTL. In other words, two different generations can be considered as two populations that have a genetic correlation between them that may be lower than unity. The results of this study may therefore partly explain the need for frequent retraining of genomic prediction models to achieve constant accuracy across generations (Sonesson and Meuwissen 2009; Wolc *et al.* 2011). We expect, however, that the change in allele frequency at a single QTL is relatively small across a few (4-5) generations, especially for traits that are highly polygenic. As a result, $r_{g}$ may be relatively high across a few generations. Nevertheless, the relative contribution of non-additive effects to the decline of genomic prediction accuracy across generations is currently unknown, and would be an interesting topic for future research.

### Conclusion

Our findings show that the genetic correlation between populations ($r_{g})$ is partly determined by the difference in allele frequencies between populations and the magnitude of non-additive effects. Large differences in allele frequencies and large non-additive effects resulted in low values of $r_{g}$. For both dominance and epistasis, when non-additive effects become extremely large, $r_{g}$ has a lower bound that is determined by the nature of non-additive effects, and the difference in allele frequencies between populations. In addition, we found that with epistasis, $r_{g}$ depends on the level of total functional epistatic variance, which is a function of epistatic effect size and the number of interactions per locus. Given that dominance variance is usually small and there is not much overdominance, we expect that it is unlikely that values of $r_{g}$ below 0.8 are due to dominance alone. With supposedly realistic epistasis, $r_{g}$ could be as low as 0.45. These results may contribute to the understanding of differences in genetic expression of complex traits between populations, and may help in explaining the inefficiency of genomic prediction across populations.
